# Is it ‘cancer prevention’ or ‘risk reduction’? #Wordsmatter

**DOI:** 10.1007/s10552-021-01470-w

**Published:** 2021-07-21

**Authors:** Desiree A. H. Walker, Mary Beth Terry

**Affiliations:** 1 Columbia University Herbert Irving Comprehensive Cancer Center Community Advisory Board, New York, NY 10032 USA; 2 Department of Epidemiology, Mailman School of Public Health, Columbia University, 722 West 168th Street, New York, NY 10032 USA; 3 The Herbert Irving Comprehensive Cancer Center, Columbia University Medical Center, 1130 St. Nicholas Avenue, New York, NY 10032 USA

**Keywords:** Cancer Prevention, Cancer risk reduction, Modifiable risk, Guidelines

## Abstract

In this commentary, we examine whether we should reconsider the widespread use of the words ‘cancer prevention’ and replace them with the words ‘cancer risk reduction’. Our recommendation is because ‘risk reduction’ more accurately reflects what we know from cancer research, but more importantly recognizes the confusion and potential harm to patients from the inaccurate use of the words ‘cancer prevention’.

## The view from a cancer researcher

The landmark epidemiological study by Doll and Peto supporting that most cancers are ‘preventable’ [1] was published almost 40 years ago; its findings have largely stood the test of time [2]. The major gains in reduced incidence at a population level have been dramatic, particularly for smoking-related cancers. However, some cancers continue to increase particularly in adults under 40 years [3]. If most cancers are preventable, why have we failed in preventing more cancers? One reason may be a failure to translate the findings. An essential part of translation involves clear language. In public health, prevention is routinely understood to apply at the population level and that it may not apply to all individuals. For most chronic diseases, as well as infectious diseases without effective vaccines and high vaccination coverage, we are never able to fully prevent an outcome at an individual level even if we can reduce risk at a population level. Both the Human Papilloma Virus and Hepatitis B vaccines hold tremendous promise for major declines in virally-driven cancers, but these vaccines are an exception as most cancers do not have a vaccine.

## Risk reduction language over prevention emphasizes that it is never too late

Although public health prevention is applied at a population level, the challenge for cancers, as opposed to many acute health outcomes, is the long induction time between exposure and disease onset. For example, many populations cannot prevent exposures to risk factors that may be theoretically modifiable (and therefore understood as preventable) but have occurred already. Thus, an emphasis on risk reduction rather than prevention is important given the long latency of most cancers. In the USA, many may only hear about risk factors for cancer in midlife, closer to the time when most cancers are diagnosed. With the exception of smoking, knowledge of other cancer risk factors is much more limited. Thus, the impact of hearing about modifiable factors and ‘cancer prevention’ later is much more limited as many exposures that increase risk may have already happened. Many of the risk factors that influence risk of first cancer also influence outcomes after a cancer diagnosis including the risk of second cancers and other health outcomes after diagnosis. Thus, moving away from the language of ‘cancer prevention’ toward the language of ‘cancer risk reduction’ across the cancer control continuum may also influence population health through risk factor modification after diagnosis.

## Risk reduction language over prevention is also important for those at higher risk based on genetic susceptibility

Risk reduction, rather than prevention, is also more useful language in considering more recent advances in genomics. Although the genomic revolution has led to a debate on ‘prevention’ versus ‘luck’ [4]; the reality of large-scale twin studies have supported that monozygotic twins only have an approximate 30% chance of getting cancer if their twin has cancer [5]. Increasingly, epidemiological data support that many modifiable risk factors are also associated with cancer risk in individuals with germline mutations in cancer susceptibility genes [6]; thus, risk modification is important even in individuals at highest risk from germline mutations even if prevention is not, and absolute risk reduction is greatest in populations at higher risk.

## Risk reduction language over prevention recognizes cancer health inequities

The burden of cancer remains unequally distributed across communities. This is where we believe the language of prevention can be even more harmful as it does not recognize existing health inequities that contributed to poorer cancer outcomes that are dramatically different in communities of color and low-income communities. Thus, given the multitude of drivers of cancer health inequities from unequal access to care to higher exposure to environmental carcinogens, the words ‘cancer prevention’ are not accurate for individuals who had no choice in where they lived, worked, and played. It is possible for everyone to reduce their risk, but it is not possible for everyone to equally prevent their cancer. This is true at an individual and a community level.

## What we can do now

Investment in research to identify new modifiable factors of cancers at both individual and community level. It is particularly important to research the drivers of early onset cancers that are rising dramatically. While this research is ongoing, we believe that significant strides can be made through greater translation of the established risk factors earlier in life and even more immediate by shifting language from prevention to risk reduction. Changing community, state, and national guidelines on cancer prevention to cancer risk reduction is more than just semantics. It is based on the science which recognizes that each cancer is a combination of factors within and beyond our control. On the clinical side, the language has already shifted to recognize that even surgical options like mastectomies and oophorectomies do not prevent cancer 100% and therefore language over the past decade has shifted from prophylactic surgery to risk-reducing surgery. Risk reduction programs, rather than cancer prevention programs, also recognizes that many changes can be made to reduce a person’s own as well as their family and community risk of cancer as well as outcomes after a cancer diagnosis.

## The view from a cancer patient advocate

### Did you know that between January and November each year 30 types of cancer are observed during their respective cancer awareness month?

Since 2004, National Cancer Prevention Month has been observed in February [7, 8]. As described when introduced in the U.S. Senate resolution, the purpose of the Cancer Prevention Month was to “take this opportunity to educate one another on the steps they can take to prevent cancer” [7, 8]. Many cancer prevention awareness campaigns include information about reducing many cancer risk factors including sun exposure, smoking, alcohol as well as information about increasing cancer screening, healthy eating, and exercise [7, 9–11].

### Did you know the cancer education months actually started 80 years ago?

Yet, national efforts to bring awareness to cancer control started much earlier when President Franklin Delano Roosevelt declared April to be Cancer Control Month in 1940, and said “It is an opportunity to educate the public about the need to prevent cancer through public health initiatives such as screening and to improve access to care and treatment” [7]. At this same time, the American Cancer Society under the former name of the American Society for the Control of Cancer also promoted Cancer Control Month campaigns [7, 12]. Together cancer awareness campaigns are supported across many governmental organizations including the Centers for Disease Control and Prevention [13] and the National Cancer Institute (NCI) [14]. Cancer Awareness campaigns seem straightforward.

### Where do people seek definitions from?

Merriam-Webster Lerner’s Dictionary defines prevention as the act or practice of stopping something bad from happening: the act of preventing something [15]. The NCI defines prevention in medicine, as action taken to decrease the chance of getting a disease or condition [16, 17]. If a poll was given to the general population asking what do they believe ‘cancer prevention’ is, their interpretation would be derived from Merriam-Webster. Most people believe that their actions would either stop cancer from occurring or promote cancer to occur. It is safe to say that the NCI’s definition is generally unknown within the general population and the messaging of the numerous cancer campaigns have not increased the knowledge around what one’s behavior/lifestyle can be expected to do or not do.

### How do we interpret evidence-based research?

Today, research has informed us that not all cancers are preventable. Therefore, the blanket statement that cancer can be prevented conveys an inaccurate message and creates a false sense of reality. If you are one of the 1.8 million people expected to receive a cancer diagnosis in the USA in 2020 [17], who followed the recommendations, you will be baffled. It is also worth noting, the World Health Organization says that only 30–50% of all cancer cases are preventable [18]. Given this fact, the real question is why is ‘cancer prevention’ frequently stated without the specifics regarding which cancer cases fall into the 30–50% category.

### Breast cancer is one of the most common cancers in American women. Can it be prevented?

As someone who has experienced breast cancer twice, I am now one of the more than 3.5 million women with a history of breast cancer in the USA [19]. Hearing or reading the words ‘cancer prevention’ is disconcerting since breast cancer is currently not preventable except for taking certain medications that may reduce risk for women at risk of hormone-receptor positive cancer. Oh and of course we are told we can have a risk-reducing mastectomy. Most women would not view either medications with side effects or surgeries as prevention. The truth is the lifestyle changes often mentioned are the cancer risk factors that can be controlled. Our behaviors can help lower breast cancer risk but do not guarantee the words “You have breast cancer.” will not be heard. As much as lifestyle changes are emphasized, it would be important to advise that age, sex, race or ethnicity, family history, and inherited genetic characteristics are cancer risk factors that are uncontrollable [10]. It is true that about five to ten percent of breast cancers are caused by an inherited genetic mutation, with the remainder of cancer risk factors accountable to unplanned changes that occur during an individual’s lifetime [10].

To talk about the uncontrollable cancer risk factors is not so simple. Yes, it is like opening Pandora’s box. Did you know that body weight could be influenced by genetics, environment and/or behavior? [10]. If you are a smoker, you now have environmental exposures so quitting is an option to address this [10]. Have you heard there are other environmental exposures such as parabens, triclosan, phthalates, plastics, and lead that lead to pollutants, contamination of natural environments and significant health issues? If you were not aware, then it is safe to say that you have already been exposed. While we look forward to the day that breast cancer is preventable, until then the newly diagnosed, survivors, thrivers, and those living with cancer recommend the use of ‘risk reduction’ in lieu of ‘cancer prevention’ in the communications from and vernacular of those in the public health and oncology fields so there is no ambiguity.

### Does the phrase ‘cancer prevention’ empower or do harm?

Other cancer survivors would describe their experience when they heard the words “You have cancer.” as jarring and a gut punch too, especially when there was no family history of the disease! Many would agree with the sentiments shared that hearing ‘cancer prevention’ gives a false assurance. Though those that use ‘cancer prevention’ may want to convey hope, it actually creates emotional upheaval when a diagnosis is received. False assurances do not allow a person to be realistic and/or prepared for all possibilities. Everyone deserves honesty.

### Which stage of the life course should risk reduction be incorporated?

Actually, lowering one’s cancer risk should start in early childhood. The Centers for Disease Cancer Prevention and Control have research indicating healthy behaviors may lower cancer risk and how avoiding harmful exposures can protect the health of babies, young children, youth, and young adults. Thus, reducing the chance of getting cancer in the future [20]. The consequences of health behaviors and harmful exposures start to arise during mid-life [21]. However, adults who experience health challenges should make every effort to address their health behaviors and avoid harmful exposures to support better health today and beyond [21].

### What are the key takeaways?

Since President Roosevelt wanted to improve access to care we must acknowledge that one’s socioeconomic status is linked to cancer too [10]. Lest we disregard that the type of work a person does, stress levels, and access to the health care system play a role in overall health [10], but for this article we will solely focus on the cancer-related risk factors. Perhaps you will agree what started out straightforward with cancer awareness months, a National Cancer Prevention Awareness Month and a Cancer Control Month became complex when we talked about controllable and uncontrollable risk factors for cancer and included the impact of socioeconomics on access to healthcare. It is safe to say that risk reduction is actionable, albeit complex. If we agree that all cancers cannot be prevented, it is the hope that the use of ‘cancer prevention’ is reconsidered since it has been outlined that it causes more harm because of the false assurance it creates. Utilizing ‘risk reduction’ in the public health campaigns and conversations will inform the general population how fostering healthy behaviors can lead to better health outcomes.
